# Supplementary material for: Multi-Omics Approach to Mitochondrial DNA Damage in Human Muscle Fibers
Source: Int J Mol Sci. 2021 Oct 14;22(20):11080. doi: 10.3390/ijms222011080 (PMC8537949; doi:10.3390/ijms222011080)
Supplement: Supplementary file 1 [file ijms-22-11080-s001.zip › Table S1.pdf]

Table S1. Characteristics of the patient cohort and corresponding raw file list.

Patient N	Patient ID	Muscle	Nuclei	Sex	Age at Biopsy	mtDNA	COX section pool	ILM_GENE file
1	032/05	biceps	<10% activated subsarcolemmal nuclei, but not central	f	71	POLG, no document. on mtDNA deletions	COX-	X4286624097_A
							COX+	X4286624097_B
2	039/07	triceps	nuclei subsarcolemmal not centrald	m	54	sd	COX-	X4286624097_C
							COX+	X4286624097_D
3	391/06	biceps	nuclei subsarcolemmal few central	f	31	sd	COX-	X4286624097_E
							COX+	X4286624097_F
4	263/01	deltoid	partly activated subsarcolemmal nuclei, few central	m	54	multiple mtDNA deletions, OPA1 variant	COX-	X4286624076_A
							COX+	X4286624076_B
5	191/02	vastus lat	not described	f	41	sd	COX-	X4286624076_C
							COX+	X4286624076_D
6	392/07	nk.	nuclei subsarcolemmal not central	f	42	sd	COX-	X4286624076_E
							COX+	X4286624076_F
7	469/07	biceps	increased central	f	43	sd	COX-	X4286624074_A
							COX+	X4286624074_B
8	008/09	biceps	little increased central	m	68	multiple mtDNA deletions	COX-	X4286624074_C
							COX+	X4286624074_D
9	584/08	biceps	increased central	m	59	sd	COX-	X4286624074_E.
							COX+	X4286624074_F

Sd, single deletion. Nk not known
